# Frequency of intra-abdominal organ injury is higher in patients with concomitant stab wounds to other anatomical areas

**DOI:** 10.1186/s12873-018-0167-4

**Published:** 2018-06-27

**Authors:** Igor Jeroukhimov, Itay Wiser, Yehuda Hershkovitz, Zahar Shapira, Kobi Peleg, Ricardo Alfici, Adi Givon, Israeli Trauma Group, Hany Bahouth, Alexander Becker, Michael Ben Ely, Alexander Braslavsky, Igor Jeroukhimov, Milad Qarawany, Boris Kessel, Yoram Klein, Guy Lin, Ofer Merin, Miklosh Bala, Youri Mnouskin, Avraham I. Rivkind, Gad Shaked, Dror Soffer, Michael Stein, Michael Weiss, Boris Kessel

**Affiliations:** 1 Trauma Unit, Assaf Harofeh Medical Center, Zerifin 7030000, Israel, affiliated with the Sackler Faculty of Medicine, Tel Aviv University, Tel Aviv, Israel; 2 National Center for Trauma and Emergency Medicine Research, Gertner Institute for Epidemiology and Health Policy Research, Sheba Medical Center, 52621 Tel Hashomer, Israel; 3 Surgical Division, Hillel Yaffe Medical Center, 38100 Hadera, Israel; 4 Trauma Unit, Hillel Yaffe Medical Center, 38100 Hadera, Israel

**Keywords:** Stab wound, Penetrating abdominal organ injury, Abdominal trauma

## Abstract

**Background:**

Management of stable patients with abdominal stab wound remains controversial, particularly for those with no clear indications for explorative laparotomy. We evaluated the risk of intra-abdominal injury in stab wound victims concomitantly stabbed in other anatomical body areas.

**Methods:**

We performed a retrospective cohort study of patients with abdominal stab wounds recorded in the Israeli National Trauma Registry from January 1st, 1997, to December 31st, 2013. Patients with an isolated abdominal stab wound were compared to those with concomitant stab wounds to other anatomical areas. Intra-abdominal organ injury was defined by imaging or surgery findings. Multivariate analysis using a logistic regression model was conducted to assess independent risk for intra-abdominal organ injury.

**Results:**

The study included 3964 patients. After controlling for age, gender and hypotension on arrival, patients with multi-regional stab wounds had an increased risk of intra-abdominal injury (OR = 1.3, CI 95% 1.1–1.6, *p* < 0.001). These patients also had a higher rate of injury to the solid organs than patients with an isolated abdominal stab wound.

**Conclusions:**

Patients with multi-regional stab wounds have an increased risk of intra-abdominal injury. Worldwide accepted “clinical follow up” protocol may not be appropriate in management of patients with multi-regional stab wounds.

## Background

Penetrating abdominal trauma continues to be a major cause of trauma admissions worldwide [1]. Non-operative management is a widely accepted approach for the stable patient with an abdominal stab wound who does not require urgent surgery [2–4].

Clinical observation of stable patients with anterior abdominal stab wounds with no signs of peritonitis has been adopted by most of the trauma centers [5].

Currently, the management approach for patients with multi-regional stab wounds including the abdomen is not distinguished from those with isolated abdominal stab wounds.

We assume that the management approach to the patients with multiple abdominal stab wound may be different from those with isolated abdominal stab wound. That was intuitively based on idea that multiple stab wounds have potentially worse mechanism of injury and higher probability to penetrate peritoneal cavity and cause more damage. We hypothesized that the rate of intra-abdominal injury is higher in patients with multi-regional stab wounds compare to patients with isolated abdominal stab wounds.

Our aim was to find out whether an association exists between intra-abdominal injury and other anatomical area involvement in stab wound injury.

## Methods

### Study population

We conducted a retrospective cohort study of trauma patients with abdominal stab wounds admitted to 19 Trauma Centers between the years 1997 and 2013. Data was obtained from the records of the Israeli National Trauma Registry maintained by Israel’s National Center for Trauma and Emergency Medicine Research, Gertner Institute for Epidemiology and Health Policy Research. This institute records information concerning trauma patients hospitalized in 19 hospitals of which six are Level I, and 13 are Level II Trauma Centers. Data was obtained from the Israeli National Trauma Registry and included only anonymous information regarding the patients with no possibility of identification for the purposes of quality assessment and research. Ethical approval was obtained from the Helsinki Committee at Sheba Medical Center where the Israeli Trauma Registry located (Approval Number 5138–18-SMC).

The study population was divided into two groups: isolated abdominal stab wounds and abdominal stab wound with concomitant multi-regional stab wounds (defined as one or more concomitant stab wounds located in an anatomic area other than the abdomen). Knife was the only assaulting instrument. Patients with isolated thoraco-abdominal stab wound and patients with multiple abdominal stab wounds were excluded from the study.

Data collected included: age, gender, presence and location of intra-abdominal injury according to ICD-9 diagnosis, Injury Severity Score (ISS), Abbreviated Injury Score (AIS), systolic blood pressure on admission, Glasgow Comma Scale (GCS), hospital length of stay (LOS) and mortality.

Patients with admission systolic blood pressure 90 mmHg or less were defined as hemodynamically unstable. In case of hemodynamic instability, presence of peritoneal signs, or evisceration of abdominal content all the victims were referred directly to operating room. In absence of absolute indications for surgery, the treatment algorithm included observation with serial physical examinations, local wound exploration, computed tomography and diagnostic laparoscopy in selected cases depends of particular hospital protocol.

We compared the presence of abdominal penetration and the other variables between both groups.

### Data analysis

Statistical analysis was performed using the SAS statistical software (SAS, Cary, NC, version 9.2). Statistical tests included chi-square tests for categorical data and the Wilcoxon nonparametric test for continuous variables that were not normally distributed. A multivariate analysis was conducted using binary log two-tailed. Univariate and multivariate odds ratios appear for all variables (unadjusted – univariate, adjusted – multivariate).

## Results

### Participants

We found 438,765 trauma victims in the Israeli National Trauma Registry between the years 1997 to 2013. Of these, 3964 suffered from abdominal stab wounds (0.9%). Baseline characteristics of both groups are presented in Table 1. No significant differences were observed between groups in age and gender. The multi-regional stab wounds group had a significantly higher rate of hypotension on arrival and GCS ≤8 compared to the group with an isolated stab wound (9.3% vs 5.5%, *p* < 0.0001 and 4.1% vs. 1.3%, *p* < 0.0001; respectively). The intra-abdominal injury rate was higher in the multi-regional stab wound group of patients (40% vs. 35%, *p* = 0.0004) (Table 2).

**Table 1:** Baseline Characteristics, Injury Pattern and Outcomes ^a^

	n/n (%)			*p* Value
	Isolated ASW	Multiregional SW	All	
Demographics				
Age ≥ 50	88/1866 (4.7%)	118/2088 (5.7%)	206/3954 (5.2%)	0.19
Male gender	1786/1870 (95%)	1993/2093 (95%)	3779/3963 (95%)	0.67
Injury characteristic				
SBP < 90 mmHg	101/1852 (5.5%)	193/2072 (9,3%)	294/3924 (7.5%)	< 0.0001
GCS ≤8	24/1833 (1.3%)	83/2046 (4.1%)	107/3879 (2.8%)	< 0.0001
Abdomen AIS ≥3	443/1871 (24%)	517/2093 (25%)	960/3964 (24%)	0.45
Intra-abdominal injury	660/1871 (35%)	852/2093 (40%)	1512/3964 (38%)	0.0004
ISS ≥25	19/1871 (1%)	197/2093 (9.4%)	216/3964	< 0.0001
Outcomes				
Mortality	17/1871 (0.9%)	53/2093 (2.5%)	70/3964 (1.8%)	0.0001
LOS, mean (SD)	3.9 ± 5.4 (*n* = 1866)	5.2 ± 6.5 (*n* = 2077)	4.6 ± 6.0 (*n* = 3943)	< 0.0001

*SW* stab wound, *ASW* abdominal stab wound, *SBP* systolic blood pressure, *GCS* Glasgow Coma Scale, *AIS* Abbreviated Injury Score, *ISS* Injury Severity Score, *LOS* hospital length of stay

^a^ Categorical variables were compared using Chi-square test, Continuous variables were compared using Wilcoxon rank test

**Table 2:** Comparison of Penetration/Penetration with Intra-abdominal Injury Incidence in Patients with Isolated Versus Multiple Anatomical Area Stab Wounds

Type of injury	Abdominal and other area (*n* = 2093)	Abdominal only (*n* = 1871)	Total (*n* = 3964)	*p* Value
No penetration	693 (33.1%)	568 (30.5%)	1261	0.063
Penetration only	548 (26.2%)	643 (34.4%)	1191	< 0.001
Penetration with intra-abdominal injury	852 (40.7%)	660 (35.3%)	1512	0.0004

Mortality was significantly higher (2.5% vs. 0.9%, *p* = 0.0001) and mean LOS was longer in patients with multi-regional stab wounds (5.2 ± 6.5 vs. 3.9 ± 5.4 days, *p* < 0.0001). Frequency of injury to the liver, kidney and spleen was higher in the multi-regional stab wound group (13.2% vs. 8.4%, *p* < 0.0001; 5.6% vs. 3.6%, *p* = 0.004; 8.2% vs. 1.2%, *p* < 0.0001; respectively). The small bowel was injured more frequently in the isolated abdominal stab wound group (10.4% vs. 5.4%, *p* < 0.001) (Table 3).

**Table 3:** Comparison of Intra-abdominal Injury Abdominal Stab Wound Patients with or without Other Area Involvement

Organ	Abdominal and other area (n = 2093)	Abdominal only (n = 1871)	*p* Value
Vascular	73 (3.5%)	80 (4.3%)	0.2
Liver	276 (13.2%)	158 (8.4%)	< 0.0001
Kidney	117 (5.6%)	68 (3.6%)	0.004
Spleen	171 (8.2%)	36 (1.9%)	< 0.001
Stomach	92 (4.4%)	77 (4.1%)	0.66
Pancreas	16 (0.8%)	24 (1.3%)	0.103
Small bowel	213 (10.1%)	516 (27.5%)	< 0.001
Colon/rectum	97 (4.6%)	109 (5.8%)	0.09

Independent adjusted risk factors for intra-abdominal injury were systolic blood pressure ≤ 90 mmHg (OR = 1.5 (1.1–2.1, *p* = 0.02), GCS < 13 (OR = 3.9 (2.4–6.2, p < 0.001), age ≥ 50 years (OR = 2.2 (1.01–4.6, *p* = 0.048) and multi-regional stab wounds (OR = 1.3 (1.1–1.6, *p* < 0.001) (Table 4).

**Table 4:** Univariate versus Multivariate Analysis of Risk Factors for Intra-abdominal Injury in Patients with Abdominal Stab Wounds

Variable	Unadjusted	*p*-Value	Adjusted ^a^	*p* Value
SBP ≤90 mm HG	2.24 (1.7–2.9)	< 0.001	1.5 (1.1–2.1)	0.02
GCS < 13	6.2 (4.2–9.3)	< 0.001	3.9 (2.4–6.2)	< 0.001
Age ≥ 50	3.3 (1.7–6.1)	< 0.001	2.2 (1.01–4.6)	0.048
Female gender	1.24 (0.9–1.7)	0.16	1.16 (0.8–1.7)	0.45
Multiregional stab wounds	1.24 (1.1–1.4)	0.001	1.3 (1.1–1.6)	< 0.001

*SBP* systolic blood pressure, *GCS* Glasgow Coma Scale

^a^ Regression odds ratio compared with: systolic blood pressure 90 mmHg and above, GCS 15, age group 0–14 years, male gender, abdominal-only stab wounds

### Key results

Patients with multi-regional stab wounds have a higher rate of intra-abdominal injuries compared with patients with isolated abdominal stab wounds.

## Discussion

Until the late twentieth century, operative management was the only accepted standard for penetrating wounds to the abdomen. In 1969, in a study of 600 stabbed patients, Nance et al. [6] reported the advantage of a selective non operative approach compared to mandatory explorative laparotomy. Later, several studies supported evidence that not all penetrating abdominal wounds require surgery. Navsaria et al. [7] in a study on 186 stabbed trauma victims successfully managed 53.8% of them non-operatively**.** Friedmann [8] in a study of 108 victims with abdominal SW found that a policy of mandatory laparotomy would have resulted in a negative laparotomy rate of 70%. Renz and Feliciano [9] in a prospective observational study found a complication rate of 42% in patients who underwent non-therapeutic laparotomy for trauma. Later, a selective non operative approach was extended not only to anterior abdominal wall wounds, but also to flank and back injuries. Ocampo et al. [10] in a study of 473 patients stabbed to the posterior abdomen demonstrated a 76% success rate with conservative management. Increased liberal use of CT in such cases demonstrated a high accuracy for the presence of intra-abdominal injury and even peritoneal violation [11, 12]. In order to avoid missing serious, but initially asymptomatic injuries, several diagnostic algorithms have been suggested. These algorithms take into consideration physical examination, local wound exploration, diagnostic peritoneal lavage and/or imaging. Eastern Association for the Surgery of Trauma guidelines strongly recommend considering abdominal CT in patients with abdominal stab wounds that have been selected for conservative management [5]. Nevertheless, potential risk of exposure to ionized radiation causing by CT should not be ignored [11, 13]. Inaba et al. [14] prospectively compared CT against serial physical examination in the evaluation of 249 patients with a stab wound to the abdomen. In their study, CT findings did not alter clinical decision making. The sensitivity and specificity of physical examination were 100.0 and 98.7%, respectively, while those of CT were 31.3 and 84.2%, respectively. The authors concluded that a physical examination-based diagnostic algorithm is effective and decreased the radiation burden in the management of patients with abdominal stab wounds.

Little is known about the therapeutic approach in the management of patients with multiple stab wounds in different body areas including the abdomen compared with isolated abdominal wounds. We live in a world where crime and the severity of violence consistently worsen over the years. Kessel et al. [15] in a study based on the national trauma database showed a gradual increase in both the absolute rate and the relative incidence of serious stab injuries in Israel. The authors also found that 50% of the victims suffered from multiple stab wounds to different body areas. A similar tendency has been reported in other countries [16, 17].

In the current study, we assumed that the frequency of intra-abdominal organ injury in patients with multi-regional stab wounds may be higher than that reported in the literature for patients with isolated abdominal stab wounds. We hypothesized that multiple stabs reflected increased aggression by the attacker, that would resulted in increased severity of the injuries. About 30% of our study population had no penetration into the abdominal cavity. Two thirds of all stab wounds patients in our study had violation of the abdominal cavity with or without intra-abdominal organ injuries. Similar findings have been reported in other studies [18, 19]. However, the major finding of this work was a significantly higher incidence of most intra-abdominal organ injuries in patients with multiregional stab wounds.

We were unable to find similar studies in the relevant English medical literature According to the literature, the most commonly injured organs in patients with stab wounds are the liver and small bowel. Each of them comprises 30% of all intra-abdominal injuries [20]. In our study, the overall incidence of solid organ injury was lower, but frequency these organs injury was significantly higher in patients with multiple body areas stab wounds.

Clinical observation and serial abdominal examination is a worldwide accepted approach to the patient with isolated abdominal SW [14]. Nevertheless it may not be appropriate in patients with multiple stab wounds due to relatively high probability of missing intra-abdominal injury.

This tendency may have an impact on current acceptable management approach to the patient with abdominal stab wound. Presence of additional to abdomen stab wounds may complicate the patient manage because of various aspects. Other wounds, even superficial, may be the source of significant pain that often requires more pain medications, sedation or in some cases even intubation. Major wounds may draw the physician attention and seem to be of major priority, thereby delay treatment of life-threatening injuries. Moreover, such injuries make the clinical observation and serial examination very limited or even impossible in some cases.

## Study limitations

The trauma registry does not include patients discharged from emergency departments. Therefore, the real incidence of non-penetrating abdominal stab wounds is unknown. However, in these mildly injured patients, the presence of other anatomical areas wounds has no impact on the abdominal management protocol.

The second limitation of this study is that we had no complete information regarding injuries aside from the abdomen. Nevertheless, we assume that the variability between the groups should be minimized by the large sample size provided by the registry.

## Conclusions

The incidence of almost all intra-abdominal organ injuries in patients with multi-regional stab wounds was significantly higher compared to isolated abdominal stab wounds. These findings may support the development of different diagnostic approaches to patients with multiple body areas stab wounds. Based on the study results we conclude that only clinical observation may not be an appropriate management approach in patients with multi-regional stab wounds. We believe that abdominal CT in patients with abdominal and other anatomical body areas stab wounds should be considered. Future prospective studies will assist to develop unique management protocol for these patients.

## Abbreviations

AIS: Abbreviated Injury Score
CT: Computerized Tomography
GCS: Glasgow Comma Scale
ISS: Injury Severity Score
LOS: length of stay
SBP: Systolic Blood Pressure
SW: Stab Wound
